# Relation between Health Literacy Levels, Hypertension Awareness and Control among Primary-secondary School Teachers in Turkey

**DOI:** 10.3934/publichealth.2017.4.314

**Published:** 2017-06-30

**Authors:** Gülay Yilmazel, Fevziye Çetinkaya

**Affiliations:** 1 Department of Public Health, School of Health, Hitit University, Çorum, Turkey; 2 Department of Public Health, Faculty of Medicine, Erciyes University, Kayseri, Turkey

**Keywords:** health literacy, hypertension, awareness, blood pressure monitoring, school teachers

## Abstract

**Objective:**

Health literacy plays a critical role in chronic disease self-management. This study aimed to determine the relation between health literacy levels, hypertension awareness and control among primary-secondary school teachers in Turkey.

**Materials and Methods:**

This descriptive and cross-sectional research was conducted among school teachers with the participation of 500 volunteer teachers of both genders. The response rate was 86.2%. To determine health literacy levels, the Newest Vital Sign Scale and Blood Pressure Concept Test were used.

**Results:**

The mean scores of all individuals were 2.12 ± 1.82 over six points although this showed “limited” levels of health literacy. The mean scores obtained from the scale were 2.13 ± 1.83 in non-hypertensives, while it was 2.06 ± 1.77 in hypertensives. Nonetheless, disease knowledge and awareness were low in teachers. Adequate health literacy levels were low according to disease awareness and control. The measured health literacy levels of teachers didn't overlap with their own assessments about health literacy skills.

**Recommendations:**

Limited health literacy levels in educators gave an impression that our education system was poor in terms of health education programs.

## 1. Introduction

Health literacy is a wide spectrum of skills and a competency that has considerable impact on health care processes to develop, comprehend, evaluate and use health information and concepts [1]. There has been a link between health literacy levels and the prevention some of the risk factors for non-communicable diseases [2]. Hypertension is a serious warning sign and closely related with cardiovascular outcomes, and which addresses urgent lifestyle changes for blood pressure control [3]. Health literacy is a strong indication to maintain control of blood pressure, adherence to therapy and self-management of disease [4],[5]. Studies have drawn attention to the association between limited health literacy and problematic patient tasks such as compliance to medical treatment, diet, regular medical controls and lifestyle changes [6]. Hypertension knowledge provides an important opportunity to advance the understanding of blood pressure measurements, risk factors and complications, and necessary lifestyle changes and goals of treatment. Health education in childhood can serve as a keystone for health literacy during adulthood, so teachers are in the major position to start this training. However, it was reported that knowledge and awareness of chronic diseases were quite low in teachers, creating an obstacle for the management of chronic diseases in the school environment [7],[8]. Low health literacy can be an obstacle for teachers in transferring health-related messages to students and also in solving their own medical problems. The aim of this study was to determine relation between health literacy levels, hypertension awareness and control among primary-secondary school teachers in Turkey.

## 2. Materials and Methods

### 2.1. Study sample

This descriptive and cross-sectional research was conducted in 2013 in the city of Çorum, Turkey. Çorum—one of the oldest Anatolian cities and known for the valuable Hittite archaeology—is located inland in the central Black Sea Region of Turkey. It has a population of 527,220. Official records of the Provincial Directorate of Education showed that there were 21 primary and 15 secondary schools with a total of 580 teachers in the province of Çorum. In secondary schools, teachers had a specialty in teaching a specific subject (mathematics, history of literature, social science, science and technology, English language, religious and cultural science, sports, and arts). The specialist teachers were grouped as according to the specific subjects they taught. The population of the study consisted of male and female teachers in primary and secondary levels of public schools in the province. We intended to reach all of the population. But this cross-sectional study was completed by 500 volunteer teachers. The response rate was 86.2%. This study was planned in accordance with the Helsinki Declaration and approved by the Erciyes University Ethic Committee.

### 2.2. Measures

In order to obtain the data, a two-stage questionnaire consisting of 50 questions was created on the basis of recent literature which was reviewed by the researchers. The first phase of the questionnaire was prepared for all of the teachers. In this section, teachers' socio-demographic characteristics, health-disease conditions, general knowledge and awareness about hypertension were questioned. Also in this part of the survey, to measure health literacy level, the Newest Vital Sign Scale (NVS) and Blood Pressure Concept Test (BPCT) took place. The NVS, a six-item scale, was developed by Weiss et al [9] in 2005 and adjusted to Turkish by Ozdemir et al [10] in 2010. The items in the scale are directed for the skills of numeracy reading, and understanding. The scale measures reading and understanding of food labels. Information on the label of ice cream was given to the participants and six questions are verbally administered. Example question: If you usually eat 2,500 calories in a day, what percentage of your daily value of calories would you be eating if you eat one? For each correct answer, one point is given, and the sum of the points indicates the level of health literacy. A score of <4 indicates limited health literacy and >4 indicates adequate health literacy. Scale's internal consistency (Cronbach's α = 0.70) and item validity (r = 0.52, *p* < 0.01) was used, as demonstrated in the study by Ozdemir et al [10]. In the present study, the internal consistency of the scale was found to be high. BPCT was a word information test, developed by the researchers to estimate hypertension health literacy in participants. The test consisted of 30 medical items associated with blood pressure directly or indirectly. We considered the 66-medical terms of Rapid Estimate of Adult Literacy in Medicine (REALM) scale for developing the test [11]. We questioned the individuals to establish if they had heard of these concepts previously.

Then the teachers' blood pressure levels were measured. Teachers' blood pressure levels were classified according to the Seventh Report of the Joint National Committee (JNC-VII) [12]. In this study, participants who had been diagnosed by a physician with hypertension and those blood pressure levels measured at ≥140/90 mmHg were considered to be hypertensive. Among hypertensive individuals, those diagnosed with hypertension before were evaluated as “aware of the disease”; among those whose blood pressure was measured lower than 140/90 mmHg were evaluated as “adequately under control”.

The second phase of the questionnaire was directed to the teachers who had been diagnosed with hypertension by a physician; their clinical characteristics, and a variety of health behaviors and skills related to the disease management was questioned.

### 2.3. Statistical analysis

Statistical analysis was performed using the SPSS 17.0 program. Independent t test, chi-square tests or Fisher's exact tests were used for categorical variables.

## 3. Results

In the study group, 66.4% were male and 33.6% were female. 57.6% of the whole group and 51.3% of the hypertensive subjects were aged 35–49. The mean age of the study group was 42.91 ± 8.75 and in the hypertensive subjects was mean age was 48.35 ± 7.53. Of the study population 36.4% had one chronic disease at least and 15.6% of the participants had previously been diagnosed with hypertension.

Table 1 presents an overview of NVS scores. The mean of scores was 2.12 ± 1.82 in the whole group and was 2.13 ± 1.83 in non -hypertensive. In individuals with hypertension, the mean scores were 2.06 ± 1.77; it is noteworthy that this is a lower mean score than for the whole group. However, there was no significant difference between the scores of individuals' with or without hypertension.

Health literacy levels of non-hypertensive and hypertensive individuals are shown in Table 1. In the whole group, 44.0% had very limited, 29.8% had limited and 26.2% had inadequate health literacy level. In the non-hypertensive group, the rate of adequate health literacy level was 26.3% while in the hypertensive group it was lower at 25.6%. However, there was no significant difference between the health literacy levels of the groups (*p* > 0.05).

When we examined the knowledge of health literacy skills of the participants, 93.2% of the whole group stated that they were able to fill out and read the forms on their own given in health establishments, 59.2% stated that they had no difficulties in understanding the medical booklet/brochure and 93.6% stated that they always read booklets and brochures. No significant difference was found between the health literacy skills of the non-hypertensive and hypertensive groups (*p* > 0.05).

**Table 1.** NVS scores and health literacy levels in hypertensives and non-hypertensives.

*NVS Scores*	*Non-hypertensives (n = 422)*		*Hypertensives (n = 78)*		*Total (n = 500)*	
*Min-Max*	0–6		0–6		0–6	
*x ± SD*	2.13 ± 1.83		2.06 ± 1.77		2.12 ± 1.82	
	t = 0.245; *p* = 0.768					

*Health literacy levels*	*Non-hypertensives (n = 422)*		*Hypertensives (n = 78)*		*Total (n = 500)*	
	*No.*	*%*	*No.*	*%*	*No.*	*%*
*Very limited*	186	44.1	34	43.6	220	44.0
*Limited*	125	29.6	24	30.8	149	29.8
*Adequate*	111	26.3	20	25.6	131	26.2
***Total***	**422**	**100.0**	**78**	**100.0**	**500**	**100.0**
	χ^2^ = 0.044; *p* = 0.978					

**Table 2.** Hearing about blood pressure terms in hypertensives and non-hypertensives.

*Terms*	*Non-hypertensives (n = 422)*		*Hypertensives (n = 78)*		*Total (n = 500)*		*χ^2^/p*
	*No.*	*%*	*No.*	*%*	*No.*	*%*	
*Dose*	367	87.0	66	84.6	433	86.6	0.314/0.575
*Complication*	310	73.5	62	79.5	372	74.4	1.256/0.262
*Indication*	232	55.0	37	47.4	269	53.8	1.506/0.220
*Tachycardia*	162	38.4	29	37.2	191	38.2	0.041/0.840
*Maintenance*	154	36.5	29	37.2	183	36.6	0.013/0.908
*Symptomatic*	156	37.0	26	33.3	182	36.4	0.375/0.540
*Medication*	144	34.1	25	32.1	169	33.8	0.126/0.722
*Systemic*	148	35.1	17	21.8	165	33.0	5.248/**0.022**
*Vascular*	136	32.2	27	34.6	163	32.6	0.171/0.679
*Vertigo*	118	28.0	25	32.1	143	28.6	0.539/0.463
*Antihypertensive*	102	24.2	29	37.2	131	26.2	5.762/**0.016**
*Hypotensive*	96	22.7	24	30.8	120	24.0	2.322/0.128
*Diuretic*	90	21.3	21	26.9	111	22.2	1.194/0.275
*Arrhythmia*	76	18.0	17	21.8	93	18.6	0.623/0.430
*Cardiomegaly*	70	16.6	18	23.1	88	17.6	1.912/0.167
*Systolic*	58	13.7	20	25.6	78	15.6	7.077/**0.008**
*Contraindication*	61	14.5	9	11.5	70	14.0	0.465/0.495
*Hemorrhage*	57	13.5	11	14.1	68	13.6	0.020/0.888
*Diastolic*	42	10.0	16	20.5	58	11.6	7.159/**0.007**
*Papilla*	40	9.5	2	2.6	42	8.4	Fisher's/**0.045**
*Defect*	33	7.8	5	6.4	38	7.6	0.186/0.666
*Postural*	30	7.1	1	1.3	31	6.2	Fisher's/**0.031**
*Ischemia*	22	5.2	8	10.3	30	6.0	2.969/0.085
*Adverse*	27	6.4	2	2.6	29	5.8	Fisher's/0.289
*Diagnose*	21	5.0	1	1.3	22	4.4	Fisher's/0.227
*Syncope*	18	4.3	3	3.8	21	4.2	Fisher's/0.865
*Vasodilator*	14	3.3	5	6.4	19	3.8	1.723/0.189
*Retinopathy*	16	3.8	2	2.6	18	3.6	Fisher's/0.593
*Sedentary*	12	2.8	2	2.6	14	2.8	Fisher's/0.891
*Vasoconstrictive*	9	2.1	1	1.3	10	2.0	Fisher's/0.622

Hearing about blood pressure terms in the participants is given in Table 2.

In the study group, the most common terms heard regarding with blood pressure were “dose”, “complication” and “indication”. A significant difference was found between the knowledge of “systemic, antihypertensive, systolic, diastolic, papilla, postural” terms in hypertensive and non-hypertensive people (*p* < 0.05). Also, this difference was greater when applied to the hearing of “systemic”, “diastolic”, “papilla” and “postural” terms; knowledge of these being less in the non-hypertensive group than in the hypertensive group.

Adequate health literacy level according to hypertension awareness and control in participants didn't show any significance. Among the people with hypertension, adequate health literacy level was higher in those aware of the disease than those not aware (*p* > 0.05). Among the individuals who had previously been diagnosed with hypertension, adequate health literacy level was 28.3% in whose blood pressure was currently under control while it was 9.1% in whose blood pressure was not under control.

Two of the most well-known signs of the disease were headaches and palpitations among teachers [13]. Signs and complications of hypertension were known commonly by hypertensive people. In particular, there was a significantly difference between groups regarding knowledge of fatigue, sleep difficulties, burning in eyes, heart and kidney failure (*p* < 0.05).

In the study group, 97.0% of the people knew that hypertension was a major problem even though knowledge about the disease definition, risk factors and values for high blood pressure was found to be low. People without hypertension had lower knowledge and attitude about the disease than those with hypertension. However, there was no significant difference between the age groups for knowledge and attitude about the disease (*p* > 0.05).

**Table 3.** Knowledge about signs and complications of hypertension in hypertensives and non-hypertensives.

	*Non-hypertensives (n = 422)*		*Hypertensives (n = 78)*		*Total (n = 500)*		*χ^2^/p*
	*No.*	*%*	*No.*	*%*	*No.*	*%*	
*Signs*							
Headache	348	82.5	60	76.9	408	81.6	1.346/0.246
Palpitation	240	56.9	40	51.3	280	56.0	0.835/0.361
Fatigue	192	45.5	45	57.7	237	47.4	3.927/**0.048**
Mental confusion	173	41.0	39	50.0	212	42.4	2.186/0.139
Power loss	135	32.0	28	35.9	163	32.6	0.457/0.499
Nausea	106	25.1	23	29.5	129	25.8	0.656/0.418
Lethargy in limbs	104	24.6	25	32.1	129	25.8	1.887/0.170
Sleep difficulties	76	18.0	23	29.5	99	19.8	5.461/**0.019**
Burning in eyes	48	11.4	20	25.6	68	13.6	11.403/**0.001**
*Complications*							
Dizziness	248	58.8	51	65.4	299	59.8	1.199/0.274
Heart attack	222	52.6	45	57.7	267	53.4	0.684/0.408
Stroke	198	46.9	46	59.0	244	48.8	3.829/0.050
Ringing in the ears	150	35.5	31	39.7	181	36.2	0.502/0.478
Heart failure	113	26.8	31	39.7	144	28.8	5.398/**0.020**
Double vision	95	22.5	20	25.6	115	23.0	0.364/0.546
Kidney failure	70	16.6	26	33.3	96	19.2	11.900/**0.001**
High cholesterol	63	14.9	18	23.1	81	16.2	3.219/0.073
Diabetes	40	9.5	13	16.7	53	10.6	3.589/0.058
Cerebral hemorrhage	15	3.6	4	5.1	19	3.8	Fisher's/0.517

In Table 4, lifestyle-change awareness about disease was given. Hypertensive people were more aware of lifestyle changes for the disease compared to non-hypertensive. Especially, the rates of knowledge was significantly higher regarding weight control, regular exercise, not smoking, and reducing alcohol consumption (*p* < 0.05).

Also, we examined adequate health literacy levels according to skills and behaviors related to the disease management of hypertensive participants. Among hypertensive people, 26.1% with adequate levels of health literacy indicated that their relationships with physicians were good, while the equivalent figure was 25.0% in those reported that their relationship was moderate or bad. Also, an adequate health literacy level was found amongst the 25.8% who stated that they were informed about the disease by their doctors and was 26.6% in those who stated that they participated seminars and conferences on disease, while it was 25.0 % in those who reported that they were not informed and did not participate.

**Table 4.** Lifestyle-change awareness about hypertension in hypertensives and non hypertensives.

*Lifesytle-change awareness*	*Non-hypertensives (n=422)*		*Hypertensives (n=78)*		*Total (n=500)*		*χ^2^/p*
	*No.*	*%*	*No.*	*%*	*No.*	*%*	
*Reducing worry or anxiety in life*	368	87.2	74	94.9	442	88.4	Fisher's/0.050
*Cutting down on salt*	364	86.3	70	89.7	434	86.8	0.699/0.403
Losing weight	341	80.8	74	94.9	415	83.0	Fisher's/**0.002**
Exercising regularly	325	77.0	71	91.0	396	79.2	7.845/**0.005**
Eating less fatty foods	307	72.7	60	76.9	367	73.4	0.588/0.443
Measuring blood pressure	291	69.0	55	70.5	346	69.2	0.075/0.785
Cutting down on cigarette smoking	275	65.2	65	83.3	340	68.0	9.985/**0.002**
Avoiding too much alcohol	247	58.5	55	70.5	302	60.4	3.952/**0.047**
Going to doctor regularly	228	54.0	44	56.4	272	54.4	0.151/0.698
Drinking less coffee and tea	222	52.6	50	53.8	272	54.4	3.507/0.061
Reducing sugars in foods	189	44.8	42	53.8	231	46.2	2.174/0.140

## 4. Discussion

In our study, 44.0% of all individuals had very limited, 29.8% had limited and 26.2% had adequate health literacy level. The mean scores of all individuals were 2.12 ± 1.82 over six points; however this shows a “limited” level of health literacy (Table 1). Indeed, studies in our country reported that adequate health literacy levels were low in the Turkish community. In Bursa, mean scores of the NVS were 2.60 ± 0.08 in individuals and 28.1% of them had adequate health literacy levels [10]. In Ankara, it was found that 29.6% of two-year degree students had adequate health literacy levels [14]. In contrast to Turkish studies, it was reported that the NVS scores and adequate health literacy were relatively higher abroad. In Serbia [15] 55.9% of adults, in USA [16],[17] 50.0% of adults, in Australia [18] 79.0% of adults, in Japan [19] 75.5% of adults, in England [20] 61.0% of adults, in Ireland [21] 43.0% of adults and in Mississippi [22] 26.0% of adults had adequate health literacy levels.

Health literacy was supposed to be higher in people that had been diagnosed with any chronic disease, but conversely adequate health literacy levels were found to be lower in hypertensive in our study (Table 1). These results are in line with previous studies which link chronic diseases and limited health literacy [3],[6]. The mean scores obtained from the NVS scale was found as 3.00 ± 1.90 in American hypertensives [23]. In another study conducted in America, the mean scores of patients with diabetes had been found to be 2.87 ± 1.80 [16]. In Singapore [24] 45.1% of hypertensives, and in USA [25] 69.0%, had adequate health literacy levels.

In researches, it was indicated that individuals with limited health literacy, evaluate their health literacy skills as positive even though this did not reflect the reality of the situation, so that self-evaluation of individuals should not be trusted [10],[26],[27]. In this study, 93.2% of all individuals stated that they were able to read and fill out the forms given in healthcare organizations. In contrast, the rate of people for people who stated they were not able to understand medical booklets and brochures given to them by healthcare providers was high. Opinions about health literacy skills were found to be higher in hypertensives compared to non-hypertensives. In the study group, adequate health literacy levels were found to be low and yet the opinions of individuals about their health literacy skills were positive (Table1).

It was reported that individuals with limited health literacy didn't know medical terms sufficiently, so they entertained more risks for medication errors and adverse drug events [27],[28]. In the present study, we identified that a significant proportion of individuals indicated that they read the drug prescription and thought their health literacy skills were high although they had not learned the concepts about blood pressure satisfactorily (Table 2). It can be concluded that limited health literacy affects all individuals in the community in terms of disease knowledge, especially even more so in individuals with chronic diseases.

In the present study, 6.9% of participants who had not received a diagnosis of hypertension were identified as hypertensive, but were not aware of their condition. In our country, according to the Chronic Disease and Risk Factors Study made in 2011 by the Ministry of Health, the diagnosis rate of people was 8.8% for hypertension by the measurement of blood pressure for the first time; these people did not know that they were already hypertensive [29]. The results obtained in this study were seen to be close to nationwide.

Awareness of hypertension in the study group was found to be 72.8%. According to the PatenT-2 [30] results, hypertension awareness was 54.7% in Turkey. In Kenya [31], awareness of the disease was found to be 46.5%.

In the present study, hypertension control was found in 85.9% of hypertensive people. According to the PatenT-2 [30] results, adequate blood pressure control was achieved in 28.7% in Turkish hypertensives. However, adequate blood pressure control was shown in Canadian hypertensives [32] in 62.5% of cases, and in American hypertensives in 50% [33]. In this study, awareness and control of hypertension were higher than in studies from both the Turkish homeland and abroad; this was one of the positive results obtained from our study.

Patient behaviors comprising adherence to treatment, diet, exercise, and weight control are effective in controlling blood pressure [3]. However, it has been stated that inadequate blood pressure control and disease awareness in hypertensive individuals arise from the result of limited health literacy [34]. In this study, according to disease awareness and blood pressure control in hypertensive individuals, adequate health literacy levels were found to be low. Similar results have been shown in studies abroad. Adequate health literacy levels were emphasized as being low as according to awareness and to ensuring the control of blood pressure in individuals with hypertension [6],[19],[20],[24].

Individuals in the study group didn't know the signs and complications of hypertension; their knowledge and attitudes related to hypertension were found to be lower (Table 3). This study gives an impression that even hypertensives did not know the process of the disease sufficiently and could not grasp the importance of the this cardiovascular risk. Such a result may be associated with poor disease information due to limited levels of health literacy in individuals (Table 2). Studies conducted in the general population and in individuals with hypertension showed that knowledge and attitudes regarding hypertension were low, and that individuals with limited health literacy level had less knowledge about hypertension [4],[5],[24].

In the study group of individuals, levels of awareness about lifestyle changes required for hypertension were higher than the levels of knowledge, though still not sufficient. Levels of awareness were higher in people with hypertension than in those without hypertension (Table 4). Studies have shown that individuals were aware of lifestyle changes needed in hypertension, and awareness of these was higher in hypertensive individuals [4],[5],[24].

It was reported that health literacy strengthened the self-management behaviors and skills in chronic diseases by changing the information asymmetry between the patient-physician in favor of the patient's autonomy; conversely, chronic disease management was insufficient in individuals with low health literacy [35],[36]. In this study, skills and behaviors related to disease management were found to be insufficient in hypertensives. Nonetheless, adequate health literacy levels were found to be higher in those who communicated positively with their physicians and are informed by their physicians, and who participated in various activities by following developments related to the disease.

## 5. Conclusions

In our study, health literacy levels were limited both in hypertensive and non-hypertensive teachers. Also, adequate health literacy levels were low according to disease awareness and control. The measured health literacy levels of teachers did not overlap with their own assessments of health literacy skills. Teachers have a key duty of health education for training students and their family members and communities. On behalf of health promoting schools, the results call for primary care services to increase blood pressure screening among teachers and to train the staff on prevention and management of hypertension.
